# The research–policy–deliberation nexus: a case study approach

**DOI:** 10.1186/s12961-017-0239-z

**Published:** 2017-09-02

**Authors:** Camille La Brooy, Margaret Kelaher

**Affiliations:** The University of Melbourne, Level 4, 207 Bouverie Street, Carlton, VIC 3053 Australia

## Abstract

**Background:**

Decision-makers tend to make connections with researchers far too late in the game of public policy, expecting to find a retail store in which researchers are busy filling shop-front shelves with a comprehensive set of all possible relevant studies that a decision-maker might some day drop by to purchase. This linear type of relation between research and policy needs to be replaced by a more interactive model that facilitates both researchers obtaining a better understanding of policy processes and policymakers being more aware and involved in the conceptualisation and conduct of research. This paper explores the role of governance in facilitating the research–policy nexus, testing a typology of research utilisation based on Murray’s (Soc Policy Society 10(4):459–70, 2011) analysis that considers various degrees of researcher–policymaker deliberation in decision-making processes. The projects were all part of various evaluation efforts carried out by the researchers to explore the use of governance in health promotion activities.

**Methods:**

Three case studies were chosen to provide some specific examples that illustrate each level of Murray’s typology. The examples involve intersectoral health promotion collaborations that combine evidence-based research in health policy initiatives with various levels of researcher involvement. For all three projects, interview data was collated in the same way, coded thematically and analysed to consider the relationship between researchers and policymakers.

**Results:**

Comparing the three models and their applicability to health promotion interventions, it could be observed that all programmes demonstrated successful examples of research translation. Strong governance imperatives structuring relationships led to more successful outcomes, whereby research was successfully translated into a public policy initiative that also led to improved health outcomes. The key idea across all of these models was that strong governance arrangements mitigated some of the barriers evidenced by the varying degrees of deliberation and researcher involvement in processes.

**Conclusions:**

The paper demonstrates that successful research utilisation is related to strong governance agendas and that early and ongoing involvement of relevant decision-makers and researchers in the governance processes, that is both the conceptualisation and conduct of a study, tend to be the best predictors of success.

## Background

Public health research is conducted in order to create the evidence base required to furnish the available set of tools that can be used by policymakers, practitioners, programme planners and other decision-makers to improve processes and quality of care [1]. A common problem expressed by policy practitioners is the limited extent to which research findings are utilised to determine or guide actions [2]. For example, it has been pointed out that it takes an average of 17 years for research evidence to reach clinical practice [3], while the direct influence of research on governance policies – whether it be structural governance or managed care governance – is said to be insignificant [4]. Lomas discusses the precarious relationship between research and policymaking, showing how decision-makers tend to make connections with researchers far too late in the game, expecting to find “*a retail store in which researchers are busy filling shelves of a shop-front with a comprehensive set of all possible relevant studies that a decision-maker might some day drop by to purchase*” [5]. As a solution, Black argues that this linear type of relation between research and policy needs to be replaced by a more interactive mode that facilitates both researchers obtaining a better understanding of policy processes and policymakers being more aware and involved in the conceptualisation and conduct of research [4]. The challenge, however, lies in how best to harness this participation in order to adequately serve both policy outcomes as well as democratic agendas [4].

This research–policy relationship has been recognised in the literature with a greater focus on processes of ‘knowledge exchange’, that is the bidirectional flow of information between knowledge producers and knowledge users [6]. This differs from previous emphases on ‘knowledge transfer’, a unidirectional approach that “*with producers of research bringing research messages to the attention of decision makers and other potential users (‘producer-push’) or potential users of research seeking it out to inform practice, planning or policy-making (‘user-pull’)*” [6]. This in turn has led to a recognition of the need for intersectoral collaboration and partnerships so that public policy efforts are marked by an exponential increase in the number of stakeholders involved in various processes.

### Murray’s model

**Fig. 1:**
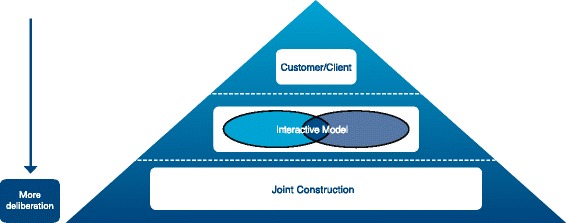
Murray's [7] typology of collaboration between researchers and policymakers

Murray [7] identifies three prevalent models that exemplify this type of intersectoral collaboration between researchers and policymakers (Fig. 1). The first model can be described as a Customer/Client relationship which corresponds to Lomas’ retail store premise, where there is a defined space for research and researchers furnish gaps in the policy process. Elliott and Popay [8] describe this relationship as a ‘problem solving’ model, whereby the policy problem is identified and the solution is sought through existing research and information is used instrumentally through a linear transfer from researcher to policymaker. This approach is often criticised for not being adequately malleable to fit to specific situations or be suitably temporally adaptable to meet with the challenges of real world policymaking. The second model, which will be called the Interactive Model for the purposes of the paper, involves some level of interaction between researchers and policymakers in a context where roles may be overlapping or where intermediaries are required to translate research outcomes. This more dialogical approach is also not without its limitations, as it remains technocratic in nature and does not encompass deliberation. In contrast, the third model is deliberatively oriented, involving a process of communication between policymakers and knowledge producers. According to Murray, in this model, “*there is a joint construction of social knowledge based on dialogue between social science and the social world, so that the process should include two-way communication between researchers, policy makers and citizens; examine the contexts in which research is to be implemented; and continue to interpret and re-contextualise the research within the implementation context*” [7]. Therefore, for the purposes of the paper, it will be referred to as the Joint Construction model. Yet, while research becomes an instrument of democratic processes, the question of how research itself can be democratised remains [7]. Nonetheless, all three models reflect the importance of maintaining a research process that is impartial, but also creative, and that can withstand the messiness of policymaking [7].

Murray’s paper attempts to bridge both deliberative and interpretative approaches in policymaking in order to enhance accessibility by both policymakers and citizens to most effectively shape deliberative policy [9]. He argues that research translation is a key factor that can improve deliberative interactions between citizens, analysts and decision-makers. Further, the incorporation of evidenced-based research in social policy allows for both an enhanced access as well as a rigorous exploration of elements of everyday life [7]. Building on Lindblom’s [10] ‘muddling through’ process and Weiss’ [11] ﻿typology that constructs seven ‘meanings’ of research utilisation, Murray synthesises the contemporary literature that examines the interactions of social scientists and policymakers, thus proposing his own typology. This typology will be applied to evaluate how deliberation takes place in three health promotion interventions implemented in the Australian state of Victoria.

As many authors indicate [7, 8, 11], guidelines on how to deal with this so-called ‘policy messiness’ as well as an exploration of how it can be overcome are absent from many accounts. The case studies explored in this analysis point to the fact that, without strong governance, relationships between research and policymakers as defined by decision-making processes are often precarious, leading to less than optimal outcomes. Thus, there needs to be greater incorporation of the literature concerning governance in analyses of research utilisation, given the fact that relationships between researchers and policymakers are defined and contingent upon various decision-making processes.

Governance is defined as the process of decision-making and the process by which resultant decisions are implemented (or not) [12, 13]. Governance encompasses actor(s), implementation processes as well as the structures established to ensure the effective implementation of the decisions [12]. Good governance in health systems promotes effective delivery of health services and population health programmes [14]. Thus, the deliberative agenda advocated by Murray goes hand in hand with that of governance goals, given that it is concerned with the distribution, exercise and consequences of power [7]. The idea of power distribution is at the heart of the deliberative method. The existence of deliberation should also involve an analysis of the exercise of power, which in health promotion interventions is evidenced by who has the ability to make decisions that are in turn structured by governance processes.

### Governance

The language of evidence-based policy – implying that there is a coherent, self-evident and uncontested body of research evidence which can (and should) be translated into policy measures – has given way to evidence-informed policy [15]. This implies a shift towards a process-based, rather than outcome-based understanding of evidence use. This language implies that policy should be made in light of relevant evidence on the issues at stake – but permits recognition of the political nature of the decision-making process in which there are competing political priorities, often with their own evidence bases [15]. Policymakers will take account of a range of other political factors (for example, stakeholder interests, available resources or institutional constraints), whilst being informed by relevant bodies of evidence. As Wehrens argues, one of the main benefits of evidence co-production is that it facilitates the redefinition and reconstruction of boundaries between the domains of policy and research purposes. Yet, the author indicates that this process lends itself to a careful “*balancing act between multiple accountability demands (which are often incommensurable and not always considered equally important) that actors within collaboration structures need to maintain*” [16]. Therefore, as it is argued, the main question to consider is not how to ‘bridge the gaps’ between research and policy, but how to investigate empirically how the domains of research, policy and practice become distinctive in some contexts and are brought together again in different ones [16]. Given this messiness, it is hard to predict ‘success factors’ of successful collaboration processes, since much of the success of a project tends to reflect programme personality. Exploring the co-production of evidence encourages analysts to more greatly reflect on the processes of demarcating and bridging boundaries between themselves and policymakers, enabling analytical focus on the strategies and actions of actors when dealing with a broad range of accountability demands.

### Aims

Murray’s [7] typology of research utilisation considers three models – the Customer/Client, Interactive Model and Joint Construction – that operationalise researcher–policymaker involvement in policy implementation. The key aim of this paper is to empirically examine this theory using health promotion initiatives in Victoria. It will explore the strengths and weaknesses of the different models outlined in the typology from the perspective of policymakers, researchers and community. It will also examine the role of governance in supporting the translation of research into policy in each model. This will both strengthen theory concerning the research–policy nexus and inform the practical application of this theory to health promotion.

As such, this paper presents a critical engagement of theory that can be used to inform implementation interventions around the use of evidence. The key aim is to explore the relationship between policymakers and researchers in health promotion initiatives and to consider its theoretical translatability to research. This is evidenced in the key examples of health promotion initiatives taking place in Victoria that adopt various methodological approaches in health policy interventions. The case studies include The Department of Human Service’s *Neighbourhood Renewal*, VicHealth’s *Prevention of Violence Against Women* (PVAW) programme and Cancer Council Victoria’s Underscreened programme. Each of the programmes represent each of the models considered in Murray’s research utilisation typology. The three exemplars involve intersectoral health promotion collaborations that combine evidence-based research in health promotion initiatives with various levels of researcher involvement. A summary of the project aims and their translatability to Murray’s models are outlined in Table 1.

**Table 1:** Summary of programme aims and research utlisation model

Programme	Aims	Research utilisation model
Closing the Gap	Divestment of power to regions to tackle Aboriginal and Torres Strait Islander disadvantage and close the gap in life expectancy through targeted interventions; Researchers provided initial population health data to inform planning efforts	Customer/Client: • Research utilisation at the outset to inform project • Researchers not involved in the Advisory Committee and had no decision-making power
Prevention of Violence Against Women	The role of researchers in the PVAW Advisory Committee was to attend meetings and to use their technical and other experience to provide feedback in relation to any of the initiatives that were being developed or implemented; The health promotion objectives were to prevent violence against women	Interactive Model: • Researchers consulted with throughout the process of planning and implementation in an instrumental way • Overlapping roles, high engagement, shared learning
Neighbourhood Renewal	This governance structure brought together the resources and ideas of researchers, residents, governments, businesses and community groups to tackle disadvantage in areas with concentrations of public housing; It promoted a deliberative forum where local participants had equal decision-making power to researchers	Joint Construction: • Deliberatively democratic, on-going participation from researchers and stakeholders, joint power sharing, high levels of transparency and accountability, participation and decision-making roles equal among researchers/stakeholders

### Closing the Gap

Closing the Gap represents Murray’s Customer/Client model of research utilisation. The programme was founded in late 2007 and implementation began in 2010, with programme offshoots still being implemented. The programme resulted from the Council of Australian Governments partnership between all levels of government to work with Aboriginal and Torres Strait Islander people to bridge discrepancies in ameliorating Indigenous disadvantage. Closing the Gap has involved a meshing of top-down and bottom-up approaches to policy development and has been viewed as a key mechanism to make policy and institutions more inclusive of citizens and more responsive to their needs. The Closing the Gap initiative represents Murray’s model as a customer/client relationship between expert knowledge and government/stakeholders. While regions had ultimate decision-making power regarding the rollout of specific plans tailored for their local jurisdictions, the population health data provided by experts to the regions ultimately informed deliberative proceedings. Experts were used instrumentally, predominantly at the planning phase, to inform regions about population data and population need and indicators were established.

### PVAW

The PVAW programme represents Murray’s Interactive Model of research utilisation. It reflects Murray’s second model, whereby there is a considerable interaction between researchers and policymakers with a high level of overlapping roles, demonstrating the convergence of research and policy through the acceptance of academics [7]. The PVAW Advisory Committee, comprised of academics, key stakeholders and government bureaucrats, was formed in order to initiate, develop and then finalise the PVAW programme. The role of the PVAW Advisory Committee was to attend meetings and to use their technical and other experience to provide feedback in relation to any of the initiatives that were being developed or implemented. As Murray [7] argues, “*these models may better reflect the ‘circuitous and tangled’ interface between research and policy* [8]*, they remain technocratic in nature, value neutral and undemocratic* [17] *and therefore do not encompass deliberative processes predicated on citizen involvement*”.

### Neighbourhood Renewal

Neighbourhood Renewal represents the Joint Construction model of research utilisation, focusing heavily on deliberatively democratic outcomes. At the outset of the intervention, a wide-scale community survey was undertaken where researchers worked in close conjunction with community members. The deliberative focus was with citizen participation in what can be described as a place-based governance initiative undertaken on two levels, namely (1) ‘joined-up government’ to focus spending in the identified areas of disadvantage; and (2) local committees comprised of agencies and residents who design and implement strategies for their area. Residents participate in locally organised activities and events, as well as the mandated form of engagement of participation via committees.

This study looks at the relationship between research and policy, firstly testing the applicability of Murray’s typology of research utilisation by examining various degrees of researcher–policymaker deliberation in decision-making processes, and secondly, examining the extent to which governance frameworks can be included to bolster Murray’s framework to better predict each of the model’s success.

## Methods

Three case studies that illustrate each level of Murray’s typology were selected to empirically test theory about the optimal pathways to evidence-based policymaking [7]. The case studies were selected based on the advice of a panel of policymakers as exemplifying systematic attempts to improve evidence translation in the context of health promotion interventions.

As the projects were structured differently, so too was recruitment for each of the evaluation efforts. Closing the Gap and Neighbourhood Renewal involved multiple sites with separate governance structures for each site that were also variable in size according to local government jurisdictions. PVAW was a state-based initiative with a single approach to governance. Closing the Gap had 188 participants from 11 sites, while Neighbourhood Renewal included 22 participants at two sites and the PVAW programme included five participants. As the design of each of the projects being evaluated was different in terms of size and scale, this corresponded to the varying numbers in sampling frame for the interviews. Interviewee number selection reflected a diversity of perspectives across the number of groups deliberating in each project in both planning and implementation. Closing the Gap had more interviewees as it had 11 deliberation groups, followed by Neighbourhood Renewal which had two groups, while the PVAW project only had one.

For all three programmes, the interview sample comprised of senior staff/key informants representing their organisation at an official level in project reference groups and who had a strong understanding of planning/implementation processes. Interviews focused on the benefits and challenges of various aspects of participation, including from both experts and other members of the governance groups, in order to ascertain whether successful engagement and deliberation led to successful project implementation. Semi-structured interviews were conducted with key informants involved in the planning and implementation of each of the projects. The questions for each of the three projects were similar and attempted to evaluate the interviewees’ level of participation, power-sharing and decision-making arrangements, governance structures, as well as other strengths and weaknesses of the planning and implementation phases. Of particular focus was the type and nature of deliberation between experts in the various stages of the planning and implementation process. The benefits and challenges of each of the various approaches were analysed and juxtaposed against project aims and a review of the literature relevant to each of the projects. Comparing the three projects, the key question assessed was how experts were engaged to best harness their talents and expertise, and encouraged participation and shared power via decision-making processes. The issue of how governance structured these arrangements was also assessed.

For all three projects, interview data was collated in the same way, coded thematically and analysed to consider the relationship between researchers and policymakers. Furthermore, common to all sites, interviews were approximately 1 hour long and the data was transcribed and analysed thematically by grouping emerging themes. Specifically, for each case study, the benefits and challenges of the model in question was analysed from the perspective of participants involved. This allowed for comparison between models in order to determine the overall strengths and weaknesses of Murray’s typology in predicting research utilisation. Thus, for each of the models, we examine the factors that led to successful planning and implementation, including key lessons.

## Results

As mentioned in the methods, the following section presents the strengths and challenges of the deliberation models of utilising researchers according to the various models employed from the perspective of participants.

### Model 1: Closing the Gap

Closing the Gap represented a Customer/Client model of researcher engagement. A key strength of the model was that it was designed to divest power from the Commonwealth to the regions, in order to engender local Indigenous participation and to facilitate decision-making processes to be largely autonomous. As such, the role of researchers was largely instrumental; they provided regions with population health data that structured initial deliberation, particularly in the planning phase. With this programme, prescribed indicators served as measures of the major social and economic outcomes that were determined would improve opportunities and standards of living for Indigenous Australians [18]. However, problematically, at a Commonwealth level, there was no programme effectiveness data provided by experts. Therefore, while Australian states had their own Indigenous affairs frameworks to adhere to with regards to reforming principles, strategy, performance and partnership coordination in order to integrate these priority areas at a macro level, ultimately, at a programme level, there were no individual benchmarks or indicators for programme effectiveness, rendering problematic evaluation outcomes. Greater deliberation and ongoing involvement with researchers would have furnished the evidence base so that programme success could be better gauged. As such, the lack of benchmark data at the programme level to indicate success implied that the population health indicators were generationally defined as programme indicators to fit within the policy cycle.

This also posed problems as many regions had existing programmes that predated the rollout of the Closing the Gap initiative. Therefore, while technocrats were used in the initial scoping process and in the consultations with community, their absence was noted from the deliberation process. While efforts were made to ensure that regions had autonomy and power to decide what was best for their local Aboriginal communities, this lack of communication at the various levels of processes ensured that there was a feeling of dissatisfaction at the types of programmes the regions had the ability to roll out within the scope of the priority areas. Further, the lack of communication tended to engender overlapping programmes and a narrower scope for targeting disadvantage. For example, if there were already programmes around smoking cessation that were capturing large sections of the region, it was felt to be a waste of funding to ask the regions to create further programmes around this priority area. As a participant advanced,

“*If I was to say there’s one thing that’s hindered us the most, is the fact that we’ve still got multiple levels of government funding Aboriginal health, and I think that is confusing the system at a really fundamental level. I think the quicker we can get to a single Aboriginal health fund in a community…and a single Aboriginal health plan for a community, the better*”*.*

Therefore, the nature of instrumental client/customer relationships at opportune moments further contributed to problematic evaluation outcomes. It was felt that there should be greater deliberation between technocrats, policymakers and community members. The Customer/Client model was felt to be lacking methodological rigour and having experts involved in deliberation and decision-making would have facilitated more appropriate programmes being selected for funding. For example,

“*I’m not sure the people at the table have all of the right skill sets to be making decisions, and that's where if you had some external technical advisors, that may add some rigour*”*.*

In addition, participants reflected that experts would have promoted deliberative proceedings that resulted in the rollout of programmes that had a more holistic approach to health and a greater focus on the social determinants of health,

“*If we’re looking at improving health outcomes for Aboriginal people, we should be looking at having education represented, housing you know, all of the players that are involved in addressing the key determinants of health*”*.*

Therefore, greater deliberation and inclusivity of researchers in proceedings could potentially have mitigated the issues evinced in evaluating the rollout of Closing the Gap.

### Model 2: PVAW programme

With the more interactive approach of research utilisation advanced in the PVAW programme, it was felt that the different mix of experience of participants enhanced discussion and feedback. This was attributed as being a leading factor in the successful delivery of programmes. From another participant,

“*The governance was very clear in terms of what the intent of the PVAW committee was, and that was to bring those entities that had a significant role to play in the prevention space, either from an academic, from an operational, from a social policy perspective, or from a service delivery perspective, coming together and debating and, not necessarily debating, but having an overview of what was going on in terms of violence prevention for women and girls. The opportunity to hear and to communicate…the issues that impact organisations to affect change was very important.*”

It was felt that the PVAW programme was hugely successful in terms of providing an opportunity for feedback and consultation from a good mix of individuals. Various initiatives were successfully implemented as part of the PVAW programme. All project partners worked in close conjunction with the funding agency and regular communication was maintained. The range of expertise was considered vital to the success of the PVAW programme. According to a respondent:

“*I think that actually worked quite well having the three types of groups involved, because it meant there was input from the different perspectives. So I remember that there was input from, sometimes there was input from academics, definitely from the state government as well as the federal government, I think which was important given that you know national plan and framework was being put in place at the time. And then also from sort of community members and service providers and things like that, that was really useful to have all of those perspectives.*”

However, while it was felt that this mix of stakeholders was beneficial, the format of the meetings could have been better designed to harness the talents of stakeholders, as some participants felt they were superfluous to proceedings. More targeted governance focusing on the structuring of this interactive form of deliberation could have mitigated this grievance, facilitating greater inclusivity of participating researchers.

### Model 3: Neighbourhood Renewal

Neighbourhood Renewal’s Joint Construction model of research utilisation implied that committees were evenly comprised of technocrats, key stakeholders and community members. This mix was thought to lead to better strategies and to lead to a more deliberatively democratic situation where policy decisions are guided by researchers but ultimately allows people affected by the outcome of those decisions to have a say. According to a participant,

“*The underlying strategy was to, through a place*[-based] *management model, connect top-down investment with bottom-up engagement of communities.*”

Neighbourhood Renewal provided a formal mechanism to bring residents and technocrats together, and contributed to agencies being seen as more responsive to resident concerns. The programme was successfully delivered and governance committees provided residents with a voice and access to agencies, which residents credited with improvements to streetscapes and crime levels. In addition, the power sharing involved in joint construction afforded by the model insured that there were benefits in terms of social capital and local democracy via deliberative forums which in turn served to increase accountability and legitimacy of local government activities. Yet, as discussed by interviewees, while there were pros in terms of increasing the deliberative democratic capacity of the policy opportunity, there was also a risk that it could weaken legitimate authoritative representational democratic processes. For example, it can be problematic when local area governance is not tied very strongly through local government channels to the decision-making process of elected representatives and there are multiple points of accountability. Another weakness spoken about was that these local area governance arrangements were not necessarily representative. There were no formal processes to ensure fair and adequate representation and it usually is built on the energy of activists in the local community. Furthermore, while efforts were made to engage as many groups as possible within the community, people who participated were “*rather a self-selecting group rather than one that can be said to represent all aspects of the community*”*.* According to a participant, this is “*not necessarily a bad thing, but it’s a risk if you don’t acknowledge the limitations of the model*”. In addition, there have been fears that top-down functions of government do not adequately meet the aspirations of bottom-up engagement. Bureaucratic inflexibility in terms of service delivery and investment models can make participation appear tokenistic, yet providing a solution to a growing crisis of legitimacy that characterises the relationship between citizens and the institutions that affect their lives. Furthermore, there is also an issue in that much of the success of the programme delivery is dependent on the personalities and capabilities of those employed to lead the implementation.

## Discussion

Comparing the three models and their applicability to health promotion interventions, it could be observed that all programmes demonstrated successful examples of research translation. The models show that research is currently not exclusively being used in a ‘retail store’ manner, with contemporary interventions demonstrating various degrees of deliberation and governance to include researchers into the planning and implementation mix. The three initiatives examined in the paper utilise research in one of the manners outlined by Murray that eventually led to programme implementation, demonstrating the nuances of research and its relationship with the policy process [7]. The implementation of the interventions and examining participant responses show how successful research utilisation should be measured by what Lomas [5] describes as the processes and not the products of policymaking.

With the Closing the Gap initiative, research was used as the basis for conducting the intervention, yet researchers/experts were absent from the advisory committee in order to provide advice about planning and implementation processes [19]. In terms of Murray’s model, it was not necessarily the inability of researchers to provide evidence that was translatable and suitably adaptable to the processes to be of use. Rather, the type of evidence that was being provided was perhaps not always the most beneficial in terms of gauging the success of the intervention. This exposes the fact that policymakers, like customers, pick and choose the evidence and research recommendations to adopt, which does not correspond with the type of methodological rigour with which researchers are accustomed, as became apparent in evaluating the effectiveness of the programme. Thus, greater deliberation and more inclusive decision-making structures and stronger governance arrangements could have facilitated smoother proceedings and better relationships between researchers and policymakers. From an evaluation perspective, the criticisms evinced by forum members reflected the fact that they felt research could be used more strategically in the policymaking process.

The PVAW programme reflects the more interactive approach outlined by Murray whereby researchers and policymakers work together and whereby roles and decision-making jurisdictions were largely overlapping [7]. The programme was well-designed insofar as governance imperatives clearly delineated roles and decision-making power was shared between participants in planning and implementation processes. The success of this project was not only the role of research at a technocratic level but also in governance processes. The interplay between formal mechanisms of governance and research translation have been interesting in terms of adjustment periods and the impact on formal decision-making. The incorporation of researchers in these processes facilitated expedient decision-making that was methodologically sound and provided opportunities for empowerment and ownership over policy beyond initial development phases. This was felt to be the most successful of the interventions in terms of researcher and overall committee membership participation.

Murray’s deliberatively democratic approach can be applied to the Neighbourhood Renewal programme [7]. This approach highlights the problematic nature of deliberative approaches to research utilisation, given the fact that deliberation takes place at community level participation. Researchers, while participating in the intervention, are not the prime target of deliberative strategies. This tends to be the case with many deliberatively focused policies, in that they transpire more at the citizen level. Thus, as Murray pointed out, the problem remains as to how best democratise research [7]. Furthermore, the fact that citizens were involved in processes did not necessarily represent a more democratic outcome, given the ability of decisions to potentially override the authority of elected officials who may have had a greater mandate than the self-selecting group participating. Therefore, rather than more democracy, this model instead represented a diversification of power so that more people were empowered within decision-making processes. Irrespective of the levels of deliberation, a key to this intervention’s success was its strong governance structure. Much of the messiness that Murray warned against was circumvented by these strong governance agendas that clearly identified roles and decision-making [7, 9]. In addition, the programme provided an effective platform to bring together different groups in governance, which in turn sparked the genesis of a number of new initiatives to address disadvantage and led to better service integration.

In summary, within Murray’s typology, each model can be seen to have specific strengths and weaknesses in how research utilisation in public health policy can be examined [7]. The first model allows policymakers to pick and choose aspects of policy that fit their needs. Yet, this approach is highlighted as problematic by Murray because research is seldom malleable to the specific context [7]. Within the Closing the Gap initiative, however, this was not necessarily the case. Neither the researchers nor the policymakers had issues with the flexibility of the research. The problematic aspect of the evaluation stemmed from the lack of inclusion of researchers in deliberative processes and the fact that research could have been used more instrumentally in order to establish indicators around programme effectiveness. The lack of decision-making power by researchers led to forum members feeling disgruntled. This supports Murray’s ultimate claim, however, that greater deliberation, moving down the typology, facilitates smoother processes [9]. This highlights the fact that model type alone is not sufficient to ascertain success. Research translation is largely contingent on the existence of strong governance structures that stipulate roles and specify decision-making power. In terms of participation, governance acted as a means of structuring intervention efforts in order to facilitate power-sharing and deliberatively democratic outcomes. This broader participatory agenda falls in line with the WHO Health For All Strategy. Thus, governance is being seen as the best way of facilitating and ensuring the ideals of these health promotion efforts. Yet, developing an evidence base around governance and research is problematic because of a lack of clear definitions of governance and the absence of a clear taxonomy of governance interventions. Governance, as an agent for change, is often invisible within the research literature. With the second model, the greater interaction and decision-making capability of researchers in deliberative proceedings, strong governance agendas and firm outlining of roles and responsibilities guaranteed the success of the PVAW programme. Therefore, despite Murray’s reservations about this model in that it may remain too technocentric, was not an issue as power sharing was evident in the PVAW committee’s governance proceedings [7]. Further, as for the deliberatively democratic approach in Neighbourhood Renewal, the strong deliberative links between participants and researchers facilitated the co-production of literature, leading to a solution to Murray’s conundrum of how to make research itself more democratic. Thus, the strong governance imperatives structuring relationships led to successful outcomes, whereby research was successfully translated into a public policy initiative that also led to improved health outcomes. The key idea across all of these models was that strong governance arrangements mitigated some of the barriers foreseen by Murray and facilitated successful planning and implementation processes.

In terms of governance frameworks, decision-making and power analysis could enhance the work of Murray. Edelman’s [9] critique of participation points to this concept as an overused term that now merely serves an ideological function of bestowing a stamp of approval in democratically inclined societies. Others share this view of an “*uncritiqued, participation in theory and practice that can help to foster a positive image*” [20] that fails to take into account the nature of participation which should be defined more as the “active *involvement of people in making decision about the implementation of processes, programmes, and projects which affect them*” [20]. For many more critical authors, anything less than active participation is an example of ‘instrumental’ participation, and cannot be thought of as participation proper because, so long as the ‘beneficiaries’ of the process are merely passive recipients, they remain a disempowered ‘object’ of the process. Hence why, according to Nelson and Wright, “*participation means … active, not passive, involvement and it should be transformative*” [20].

Transformational participation is then related to empowerment. Nelson and Wright use the term transformational participation as opposed to ‘empowerment’ because the language used to describe a process can carry different meanings in particular contexts, highlighting that, for genuine empowerment to take place, there needs to be a shift in power at both the behavioural and structural level. Therefore, deliberation facilitates this shift in power as less powerful groups actively participate in policy processes that directly affect them. In addition, in terms of structuring this decision-making, firm governance structures need to be in place, as seen within the case studies presented by this research. Thus, Murray’s typology is useful to understand and describe research utilisation in policy processes; however, as far as using this model for evaluating policy, its utility would be reinforced by greater consideration of governance and decision-making processes.

## Conclusion

This study presented the usefulness of Murray’s [7] study that considers research utilisation in policy processes. This typology can facilitate policy evaluation, specifically planning and implementation phases, given its strong focus on the relationship between policymakers and researchers and the adoption of policy and deliberative proceedings. The case studies examined in herein demonstrate that participants in these policy processes felt that deliberation at an executive and middle management level between policymakers and researchers was of strong benefit to the intervention. Enablers to a strong research–policy relationship were regular meetings, role clarity, planning and implementation momentum, partner commitment and strong lead agencies that facilitated relationships. Finding the right mix of stakeholder involvement in interventions, creating opportunities to harness their diverse knowledge base as well as managing both their expectations were challenges faced by all interventions. These types of relationships can be precarious given the number of challenges they face from each of the sectors that they seek to bridge. Issues like methodological rigour with intervention flexibility, reconciling diverse researcher and decision-maker goals and interests, practice setting unpredictability, changing priorities, available time commitment and staff workload, and varying partner research knowledge and experience all have the ability to render this relationship tenuous. Thus, the success of these types of relationships is contingent on strong governance together with open and ongoing communication. While Murray’s model can be successfully applied to fit real world examples, his typology fails to take into account governance and decision-making processes. These phenomena have been seen to be key the success of health promotion interventions.

The paper demonstrates that successful research utilisation is related to strong governance agendas and that early and ongoing involvement of relevant decision-makers and researchers in the governance processes in both the conceptualisation and conduct of a study tend to be the best predictors of success [5, 21]. This paper tested the applicability of Murray’s typology of research utilisation in three health promotion interventions in the Australian state of Victoria [9]. In evaluating the models of research utilisation within these policy contexts, it is clear that, as Lomas argues [5], success should be defined in terms of the processes, and not necessarily the products, of policymaking. In examining research utilisation in this manner, the second model that advocated overlapping jurisdictions from researchers and policymakers represented the most successful incorporation of research within a policy setting, both in terms of successful implementation as well as participant satisfaction. Murray’s study is useful to describe the levels of deliberation and nature of interaction between researchers and policymakers [7]. However, the usefulness of Murray’s model for evaluation purposes is limited without the governance frameworks built in to assess the nature and quality of deliberation. His model is beneficial to look at how research can facilitate the policymaking process and how deliberation enhances this process. The further we progress along Murray’s [7] typology signifies greater deliberation, which supports broader research on participation as evidenced by the WHO Health For All Strategy.

## Abbreviations

PVAW: Prevention of Violence Against Women
